# Temporomandibular Joint Changes Assessed by CBCT or MRI Following Functional Appliance Therapy in Skeletal Class II Patients: A Systematic Review

**DOI:** 10.3390/children13050674

**Published:** 2026-05-13

**Authors:** Gaia Lopponi, Alessio Verdecchia, Nicolò Sicca, Giulia Benedetti, Alaa Alsafadi, Teresa Cobo, Enrico Spinas

**Affiliations:** 1 Department of Surgical Sciences, Postgraduate School in Orthodontics, University of Cagliari, 09124 Cagliari, Italy; gaia.lopponi@gmail.com (G.L.); n.sicca@studenti.unica.it (N.S.); giulia.benedetti995@gmail.com (G.B.); 2 Orthodontics Division, Instituto Asturiano de Odontología, Universidad de Oviedo, 33006 Oviedo, Spain; dralsafadi@iaodontologia.com (A.A.); dracobo@iaodontologia.com (T.C.)

**Keywords:** temporomandibular joint, skeletal class II malocclusion, functional appliance therapy, magnetic resonance imaging, cone-beam computed tomography, articular disc position, condylar remodelling, temporomandibular disorders

## Abstract

**Background/Objectives**: Skeletal Class II malocclusion due to mandibular retrusion is frequently treated with functional appliances, yet their impact on temporomandibular joint (TMJ) structures, specifically the articular disc, remains debated. This systematic review aimed to critically assess quantitative morphological and positional TMJ changes (disc, condyle and glenoid fossa) evaluated with CBCT or MRI in growing skeletal Class II patients treated with functional appliances and to explore whether these changes are associated with the onset or prevention of temporomandibular disorders (TMDs). **Methods**: The review followed PRISMA guidelines and was registered in PROSPERO (CRD420251028803). Electronic searches were performed in PubMed, Scopus, Web of Science, Cochrane Library, and Embase from inception to December 2025, complemented by manual screening. Inclusion criteria comprised controlled clinical studies in patients aged 8–16 years with skeletal Class II malocclusion due to mandibular deficiency, treated with removable or fixed functional appliances, with pre- and post-CBCT/MRI quantitative TMJ assessment. Risk of bias was evaluated using RoB 2 (RCTs) and ROBINS-I (non-randomized studies); overall certainty was appraised with GRADE. **Results**: From 937 records, 8 studies met the inclusion criteria. Articular disc outcomes were reported in fewer studies: disc position/morphology was generally stable, and when changes were observed they were favourable (partial improvement/normalization in selected cases). Importantly, no included study reported new treatment-induced disc displacement or new-onset TMD symptoms at the end of treatment. Across studies, the most consistent findings concerned condylar adaptations, commonly described as anterior and/or superior positional changes and remodelling of the condyle–fossa unit. Evidence certainty was limited by heterogeneity and methodological constraints, resulting in low to very low confidence for several outcomes. **Conclusions**: Functional appliance therapy in growing skeletal Class II patients may be associated with TMJ adaptations, predominantly involving the mandibular condyle, while limited available data may suggest disc stability and no reported short-term clinical TMD onset in included controlled studies. However, due to the limited and heterogeneous evidence base, these findings should be interpreted cautiously, and well-designed prospective studies with standardized 3D imaging outcomes and longer follow-up are needed, particularly for disc-specific endpoints.

## 1. Introduction

Skeletal Class II malocclusion represents one of the most prevalent dysgnathias in the orthodontic population, affecting a significant percentage of growing patients [1,2]. Although this condition can arise from various skeletal and dental combinations, mandibular retrognathia has been identified as the predominant etiologic factor in most cases [3,4]. Consequently, the correction of this discrepancy is frequently addressed during growth with functional appliances, aiming to improve interarch relationships and facial esthetics via mandibular advancement [5]. The biological rationale underlying the use of these devices lies in their ability to alter the biomechanical environment of the craniofacial complex [6]. It is hypothesized that the forced anterior positioning of the mandible modifies the condyle’s position within the glenoid fossa, stimulating an adaptive tissue response [7,8]. Historical histological and clinical studies have suggested that such stimulation may induce remodelling of the condylar cartilage and glenoid fossa, contributing to skeletal correction [9,10]. It has also been observed that the stress distribution and morphology of the temporomandibular joint (TMJ) differ significantly in Class II subjects compared to other skeletal patterns, potentially influencing the response to therapy [11,12]. The results in the literature are heterogeneous, and the clinical significance of the changes observed on imaging remains uncertain [13]; more recent reviews have emphasized the multifactorial nature of TMDs, downplaying the role of occlusion as a direct causal factor [14,15]. Studies on mandibular function have highlighted that movement capacity varies among different skeletal groups, suggesting caution in the interpretation of functional data [16]. Consequently, the correction of Class II malocclusion has been investigated both as a potential preventive factor and as a possible risk factor for the development of TMDs, but the available evidence remains inconclusive [17,18]. Some authors have raised concerns that mandibular advancement may predispose to disc displacement or joint instability, whereas other cross-sectional studies suggest an association between Class II malocclusion and a higher prevalence of clinical signs of dysfunction [19,20,21,22]. In recent years, research on the potential effects of orthodontic interventions on TMJ components has made substantial progress due to the adoption of three-dimensional imaging approaches. The intrinsic limitations of conventional two-dimensional radiography, such as the superimposition of anatomical structures and image distortion, have historically hindered a precise quantitative assessment of bone remodelling [23,24]. The introduction of Cone-Beam Computed Tomography (CBCT) and Magnetic Resonance Imaging (MRI) has revolutionized diagnostic capabilities [25]. CBCT allows for accurate volumetric analysis of joint spaces and condylar morphology, while MRI remains the gold standard for the evaluation of soft tissues and articular disc position [19,26].

Systematic reviews published over the last two decades have highlighted significant methodological limitations in the available literature, including small sample sizes, a lack of adequate control groups, and selection bias [27,28,29]. Although recent reviews confirm that functional appliances are associated with short-term positional and skeletal changes within the TMJ, the clinical relevance and long-term stability of these adaptations, especially concerning the articular disc, remain uncertain [27,30]. Acknowledging these considerations, there is a clear need for an updated systematic review that minimizes biases and exclusively includes controlled clinical studies utilizing advanced technologies.

Therefore, the objective of this systematic review is to critically evaluate controlled studies analyzing the quantitative 3D morphological and positional changes of the TMJ (condyle, disc, and glenoid fossa) in growing patients with skeletal Class II malocclusion treated with functional appliances, with a specific focus on disc modifications and potential relationships with TMDs.

## 2. Materials and Methods

### 2.1. Protocol and Registration

This systematic review was conducted in accordance with PRISMA guidelines with a protocol registered in the PROSPERO database (ID: CRD420251028803) [31]. This review was conducted using the PICO framework, structured as follows:

Population (P): Growing patients (ages 8–16) with skeletal Class II due to mandibular deficiency, diagnosed through cephalometric (increased ANB angle and/or positive WITS value) and/or clinical parameters, without TMD or craniofacial syndromes.

Intervention (I): Orthodontic treatment with fixed or removable functional appliances for mandibular advancement.

Comparison (C): Untreated patients (control groups) and comparative pre- and post-treatment imaging.

Outcomes (O): Morphological and positional changes in the TMJ assessed via CBCT or MRI, with special focus on discal modifications.

### 2.2. Information Sources

Electronic databases searched included PubMed, Scopus, Web of Science, Cochrane Library, and Embase, from inception to December 2025. The search strategy incorporated MeSH terms and free keywords such as “magnetic resonance imaging”, “MRI”, “CBCT”, “temporomandibular joint”, “TMJ”, “functional appliance”, “orthodontic”, “activator”, “Herbst”, “Frankel”, “twin block”, and “Forsus”. The search strategy was customized for each database and complemented by snowballing (Table S1).

### 2.3. Eligibility Criteria

The following variables were extracted from the selected papers:

Included studies were prospective controlled clinical studies with a longitudinal pre–post design, comprising randomized controlled trials (RCTs) and non-randomized controlled clinical trials (CCTs), conducted on patients aged 8 to 16 years with skeletal Class II malocclusion due to mandibular retrusion (defined by increased ANB angle and/or positive WITS value). The studies had to involve treatment with functional appliances (Activator, Herbst, Frankel, Twin Block, Forsus) and comparative pre- and post-treatment imaging (CBCT or MRI), with quantitative evaluation of TMJ parameters (position and morphology of the condyle, disc position and shape, glenoid fossa morphology, signs of osseous remodelling). No language restrictions were applied. Exclusion criteria included case reports, case series, reviews, editorials, animal or in vitro studies, studies involving patients with TMD or craniofacial syndromes, studies without pre-post imaging, or those reporting only qualitative/descriptive evaluations (see Table 1).

### 2.4. Study Selection

Three independent reviewers (GL, NS, GB) screened titles and abstracts to identify potentially eligible studies. Full texts were then assessed against inclusion criteria. Disagreements were resolved through discussion or consultation with a fourth expert reviewer (ES). The Cohen’s K coefficient for agreement between the reviewers was 0.89 indicating substantial agreement [32].

### 2.5. Data Extraction

Two reviewers (GL and NS) independently extracted data from all included studies using a standardized form based on the Cochrane Consumers and Communication Review Group model and included: author and year, study design, appliance used, type of imaging used, number of treatment patients divided on patients with an active treatment and control patients, diagnostic criteria for Class II malocclusion, follow-up after active treatment and main outcomes reporting the method used for analysis, temporomandibular joint findings, such as condyle and disc changes (disc morphology, disc position, disc activity) and articular joint spaces and other principles results as clinical outcomes. Inter-rater reliability was assessed using Cohen’s Kappa, yielding a coefficient of 0.78, which indicates substantial agreement [32]. Any discrepancies were resolved through internal discussion or, when necessary, by a third reviewer (E.S.) serving as an arbiter to ensure data integrity and minimize extraction bias.

### 2.6. Risk of Bias Assesment

The risk of bias of the included studies was assessed using design-specific tools. For randomized controlled trials, the Cochrane Risk of Bias 2 (RoB 2) [33] tool was applied across its five domains: (1) bias arising from the randomization process; (2) bias due to deviations from intended interventions; (3) bias due to missing outcome data; (4) bias in measurement of the outcome; and (5) bias in selection of the reported result. Domain-level and overall judgments (‘Low risk’, ‘Some concerns’, ‘High risk’) were derived through the official Cochrane signalling-question algorithm. For non-randomized controlled studies, the ROBINS-I tool [34] was applied across its seven domains: confounding, selection of participants, classification of interventions, deviations from intended interventions, missing data, measurement of outcomes, and selection of the reported result. Domain-level and overall judgments (‘Low’, ‘Moderate’, ‘Serious’, ‘Critical’ risk of bias, or ‘No information’) were assigned according to the official ROBINS-I guidance. Two reviewers (GL and NS) independently assessed each included study; disagreements were resolved through discussion or by consultation with a third reviewer (ES).

### 2.7. Certainty of Evidence

The overall quality of the evidence was assessed using the GRADE (Grading of Recommendations Assessment, Development and Evaluation) approach [35]. The evidence was evaluated across five domains: risk of bias, inconsistency, indirectness, imprecision, and publication bias. Quality downgrades were primarily driven by a serious risk of bias across the included studies, heterogeneity in outcome assessment, limited sample sizes, and a lack of long-term follow-up. Consequently, the overall certainty of the evidence was rated as low to very low.

## 3. Results

This systematic review aims to critically assess the available literature regarding morphological and positional changes in the TMJ (specifically, discal, condylar and glenoid fossa adaptations) evaluated via MRI or CBCT in growing patients with skeletal Class II malocclusion treated with functional appliances. Modern imaging technologies such as magnetic resonance imaging (MRI) and cone-beam computed tomography (CBCT) provide high-resolution, three-dimensional quantitative data on TMJ osseous and soft tissue structures. Additionally, this review examines whether such changes are associated with the onset or prevention of temporomandibular disorders (TMDs), addressing the clinical implications of functional therapy on TMJ health. Understanding skeletal and positional changes after functional treatment is essential not only to quantify its orthopedic effects also to assess the risk of treatment-induced TMD.

### 3.1. Study Selection

**Figure 1:**
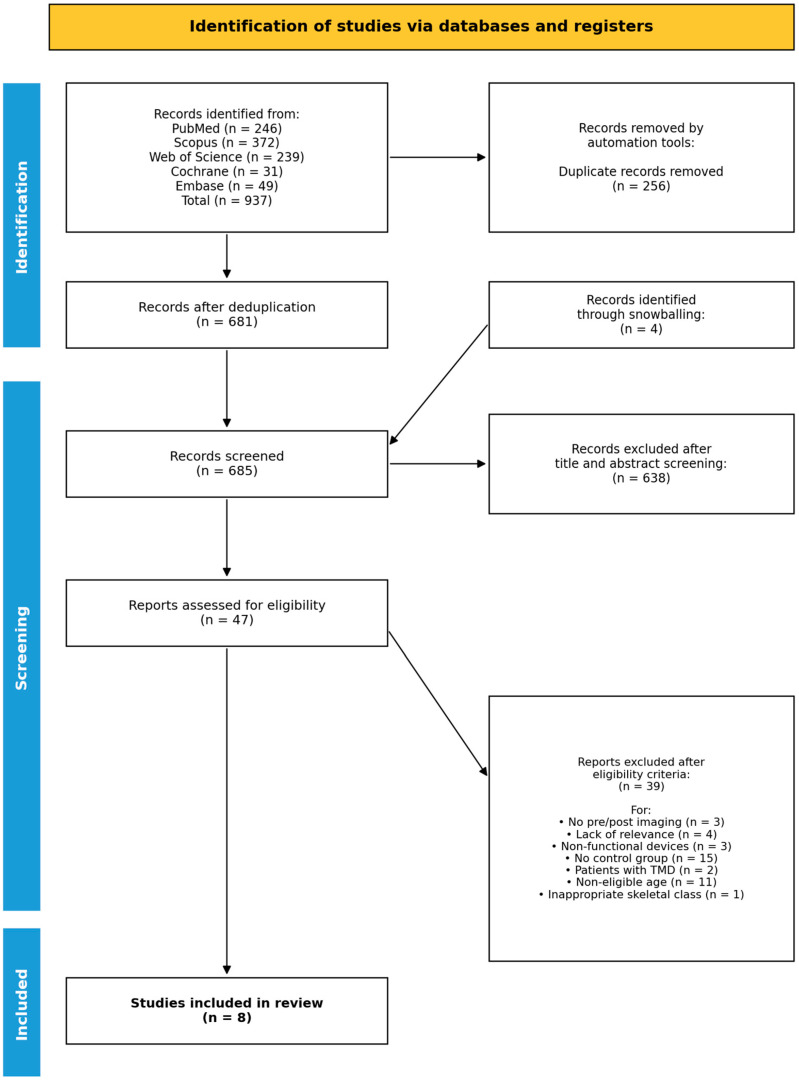
Flowchart of study selection process.

A comprehensive electronic search across PubMed (n = 246), Scopus (n = 372), Web of Science (n = 239), Cochrane (n = 31), and Embase (n = 49) initially identified 937 records. After automatic removal of duplicates, 681 records were retained for screening, to which 4 additional records identified through snowballing were added, yielding 685 records screened by title and abstract. Of these, 638 were excluded as not relevant, and 47 full-text articles were assessed for eligibility. After full-text assessment, 39 articles were excluded for the following reasons: the absence of pre- and post-treatment imaging (n = 3), a lack of relevance (n = 4), the use of devices other than functional appliances (n = 3), absence of a control group (n = 15), the inclusion of patients with temporomandibular disorders (n = 2), non-eligible age (n = 11), or inappropriate skeletal classification (n = 1). Finally, eight studies met all inclusion criteria and were selected for the systematic review. The selection process was documented using the PRISMA flowchart (Figure 1).

### 3.2. Characteristics of the Included Studies

Overall, eight controlled clinical studies met the inclusion criteria of the present systematic review, comprising three RCTs and five CCTs. The included investigations enrolled growing skeletal Class II patients (as per the review eligibility criteria: 8–16 years) with mandibular deficiency/retrusion, diagnosed through cephalometric and clinical parameters (e.g., increased ANB and increased overjet), and compared outcomes with untreated control groups. Across all included investigations, a control group was present consisting of untreated growing patients with Class II malocclusion, as specified by the inclusion/exclusion criteria. Only one study additionally included a second control group of healthy individuals with normal occlusion (Cevidanes et al., 2005) [36]. Across studies, the sample size was generally small, ranging from 18 to 78 participants overall, consistent with the controlled design and imaging-based follow-up. The functional protocols included both removable appliances (e.g., Andresen Activator, Twin Block, Bionator, Fränkel Regulator II, Twin-block) and a fixed functional appliance (Forsus). Imaging assessment was performed using MRI in most studies, while osseous 3D evaluations also relied on CBCT and CT in selected trials. Treatment duration varied from 6 to 18 months depending on appliance type and protocol, with follow-up intervals reported from 6 months up to 18 ± 1 months. A structured overview of the main methodological characteristics (study design, appliance type, imaging modality, treated/control sample, diagnostic criteria, treatment duration, and follow-up) is reported in Table 2, while Table S2 summarizes the studies analyzing the primary outcomes of our review: disc morphology, disc position and condylar position respect to glenoid fossa (Table 2 and Table S2).

#### 3.2.1. Disc and Articular Spaces Outcomes

One of the aim scopes of this work is to focalize on the disc positional and morphological changes. Between the eight included studies, only four explicitly assessed the position or morphology of the articular disc before and after treatment with functional appliances (Table S2).

Chintakanon et al., Arat et al., and Chavan et al. evaluated articular disc position, whereas Franco et al. assessed both disc position and disc morphology [37,38,39,41]. The remaining studies focused mainly on the mandibular condyle and glenoid fossa, without specific assessment of the disc. In the remaining four studies (Cevidanes et al., Elfeky et al., Jiang et al., Arici et al.), disc position or morphology was not evaluated [36,40,42,43]. These studies reported significant changes in condylar and fossa positions, primarily in the anterior and superior directions; however, no inference on disc adaptation can be drawn from these studies, as the disc was not directly assessed. None of the studies reporting disc outcomes documented the onset of new disc displacements following treatment within the assessed follow-up. Within these limits, the findings from the four studies that directly assessed the disc are consistent with the absence of treatment-induced disc displacement and, in selected cases, with morphological normalization. Overall, the findings indicate that functional orthodontic treatment in growing patients appears not to compromise disc stability and in some cases may favour improved centration or physiological morphology without short-term adverse effects.

• Disc Position

Only four of the included investigations employed magnetic resonance imaging (MRI) to directly visualize the soft tissues of the temporomandibular joint, specifically focusing on the position of the articular disc [37,38,39,41]. Consistent with the inclusion criteria requiring an absence of temporomandibular disorder (TMD) symptomatology, baseline MRI evaluations indicated that most patients possessed a physiological, asymptomatic, and superiorly positioned articular disc. Nevertheless, a small minority of the investigated samples presented with silent anterior, medial, or lateral disc displacements prior to functional intervention, specifically in Chintakanon et al., with 7.5% anterior, 5% medial, and 12.5% lateral; in Chavan et al., in all three groups, there were two cases of anterior disc displacement.; in Franco et al., partial anterior medial displacement was observed in 4 control subjects (7.1%) [38,39,41].

Following functional orthodontic therapy, none of the studies reported the induction of pathological disc displacements or internal derangements, confirming the overall safety and positional stability of the disc under orthopedic loading. However, quantitative angular assessments utilizing the “12 o’clock” method revealed a tendency for the disc to undergo a relative posterior shift in relation to the newly advanced condyle [39,41]. Chavan et al. recorded a highly significant posterior displacement of the disc relative to the condyle (*p* < 0.01) in both treatment groups, with mean sagittal angular reductions from 21.2° ± 9.3° to 1.8° ± 0.2° (a mean change of −19.4° ± 9.1°) in the Twin-Block group, and from 15.5° ± 11.6° to −0.9° ± 0.5° (a mean change of −16.4° ± 11.0°) in the Bionator group [39].

Arat et al., using a different analytical approach (measuring medial, anterior, and posterior angles relative to the condylar midline), identified slight angular variations, noting an increase in the medial angle of the disc from 56.30° ± 2.72° to 61.57° ± 4.47°, although these configurational adaptations were interpreted as physiological and did not reach statistical significance [37].

Regarding the potential for functional appliances to correct pre-existing disc displacements, the available evidence is strictly conflicting [39,41]. Chavan et al. reported that the two isolated cases of baseline anterior disc displacement documented in their sample achieved complete physiological normalization and recapture after six months of therapy [39]. Conversely, the prospective study by Chintakanon et al. found that the initial prevalence of disc displacements remained entirely unaffected by Twin-Block therapy, yielding no convincing evidence of disc recapture [41]. They reported a slight trend toward posterior disc positioning, which they attributed to differences in data acquisition between pre- and post-treatment assessments: at 6 months, in both groups (control and treated), the disc appeared to have shifted to a more posterior position, approaching the ideal “12 o’clock” reference. However, statistical analyses yielded discordant results depending on the reference line used (Frankfort plane vs. posterior condylar line). The authors concluded that this apparent posterior movement may in fact represent an artefact caused by growth-related changes in the orientation of the reference lines, rather than a true anatomical change in disc position [41]. Similarly, Franco et al., using qualitative visual assessment of the disc, did not observe any change in pre- versus post-treatment disc position with the Frankel appliance [38].

In conclusion, while functional therapy safely maintains physiological disc position and may promote favourable morphological remodelling alongside a relative posterior sagittal shift, its capacity to consistently recapture initially displaced discs remains controversial and unsupported by unanimous evidence [37,38,39,41] (Table S3).

• Disc Morphology

The morphology of the articular disc was specifically investigated only in the study by Franco et al. and, to a lesser extent, in the study by Arat et al. [37,38]. The biconcave morphology of the disc, characterized by a thinner anterior band and intermediate zone and a thicker posterior band, is clinically relevant, as it allows the disc to adapt to condylar movement and distribute functional loads. Deviations from this biconcave shape (e.g., flattened, thickened, or irregular discs) are generally interpreted as signs of structural alteration or potential pathology, often associated with displacement or temporomandibular disorders. For this reason, assessment of disc morphology serves as an indicator of its functional integrity. While Arat et al. found no statistically significant alterations, this may also be explained by the fact that the study did not strictly assess disc shape, but rather measured disc angulations (anterior, posterior, and medial) and attributed the non-statistically significant changes in the anterior and posterior disc angles to morphological adaptations of the disc, given the stability of the medial angle [37]. Franco et al., using bilateral magnetic resonance imaging after 18 months of treatment with the Fränkel II appliance, reported a statistically significant improvement in disc morphology [38]. At baseline (T1), 10.7% of discs were classified as non-biconcave (flattened or altered). Following treatment (T2), 100% of the discs exhibited a physiological biconcave morphology, with no evidence of displacement induced by therapy. These findings indicate that the articular disc remains structurally stable during and after functional treatment, and that in the minority of cases initially presenting with altered morphology, normalization toward a biconcave form can occur. In other words, Fränkel therapy not only preserves disc morphology in patients with normal baseline anatomy but may also promote significant improvement in cases with initial alteration, whereas disc position remains unchanged, consistent with the fact that all treated patients had physiologically positioned discs at baseline. In contrast, Arat et al. identified slight anatomical remodelling of the disc, which was not statistically significant and not quantifiable as a true morphological change [37]. The authors attributed these minor variations to the influence of the retrodiscal tissues and ligamentous attachments, which allow for subtle configurational adaptations without altering the true condyle-disc relationship. In summary, while the disc does not migrate within the fossa, it may undergo slight remodelling, such as changes in the inclination of the anterior and posterior bands, as a physiological adaptation to the new functional demands imposed by the appliance. This adaptive capacity allows the disc to maintain articular function without compromising structural integrity.

• Articular Spaces

Six of the eight studies included in this review (Arat et al., Chintakanon et al., Arici et al., Elfeky et al., Jiang et al., Chavan et al.) specifically examined the temporomandibular joint (TMJ) spaces, and their findings were not entirely consistent [37,39,40,41,42,43]. All of these six studies demonstrated that functional orthopedic therapies significantly alter temporomandibular joint (TMJ) spaces and induce structural remodelling, although specific adaptations vary by appliance biomechanics (TS4).

Anterior condylar repositioning was reported by studies showing anterior joint space (AJS) reductions and posterior joint space (PJS) increases:

- Arat et al., with the Andresen activator, demonstrated decreases in the AJS (*p* < 0.05) and increases in the PJS (*p* < 0.01) with no superior joint space (SJS) changes, as functional loading pressure inhibits superior growth according to earlier reports by McNamara and Woodside (Arat et al., 2001; McNamara and Woodside) [4,5,37];
- Eleky et al. with 3D cone-beam computed tomography (CBCT) showed, with the use of the Twin Block, net AJS decreases (right: −0.77 mm, *p* < 0.01; left: −0.84 mm, *p* < 0.001), PJS increases (right: +0.80 mm, *p* < 0.01; left: +1.11 mm, *p* < 0.001), SJS increases (right: 0.79 mm; left: 0.90 mm), and medial joint space decreases (Elfeky et al., 2018) [40];
- Chavan and Chintakanon et al. did not report absolute millimetre measurements but evaluated spatial changes by calculating the sagittal concentricity index with magnetic resonance imaging evaluating the sagittal concentricity index corroborated this anterior shift from a mildly anterior baseline (Chavan et al., 2014; Chintakanon et al., 2000) [39,41]; following 6 months of therapy, concentricity increased significantly (*p* < 0.01) from 3.7% to 18.7% (Twin Block) and from 7.7% to 19.1% (Bionator) (Chavan et al., 2014) [39], leaving the final condylar position significantly more anterior (*p* = 0.01) (Chavan et al., 2014; Chintakanon et al., 2000) [39,41];
- Highlighting remodelling complexity, 8-month Twin Block 3D voxel-based superimposition revealed simultaneous AJS (2.19 mm to 2.61 mm, *p* = 0.005) and PJS (2.19 mm to 3.38 mm, *p* = 0.000) increases, with altered joint space indices (−0.55 to 11.42, *p* = 0.004), driven by anterior translation alongside posterior/superior condylar apposition and fossa resorption (Jiang et al., 2020) [42];
- Conversely, computed tomography of the fixed Forsus appliance indicated posterior condylar positioning via continuous elastic forces inducing backward growth and remodelling, evidenced by volumetric AJS increases (*p* = 0.021) and PJS decreases (*p* = 0.013) compared to controls (Arici et al., 2008) [43].

• Condylar Position

The assessment of condylar position via magnetic resonance imaging (MRI) or cone-beam computed tomography (CBCT) can be performed using either the glenoid fossa or the cranial base as reference structures (Table 3). When utilizing the glenoid fossa as a reference, the analysis determines whether the condyle is centred or displaced anteriorly or posteriorly within the articular cavity. Conversely, when the cranial base or other stable structures (e.g., the nasomaxillary region or posterior cranial fossa) are employed as references, the focus shifts to the three-dimensional displacement of the condyle in space. In these cases, changes are quantified through voxel-based superimpositions or three-dimensional landmark analyses, allowing for a precise description of condylar translations and rotations (anterior, posterior, superior, inferior, or mediolateral) relative to a stable reference system. Beyond linear translations, functional therapy influences the horizontal orientation of the condyle, measured as the condylar axial angle relative to the cranial midline [41]. In untreated subjects, this axial angle underwent a statistically significant reduction over time (−4.1° ± 8.6°, *p* < 0.05) as a characteristic of the untreated Class II growth pattern, whereas in patients treated with a Twin-Block appliance, the angle remained remarkably stable (0.6° ± 9.6°) [41]. This axial stability suggests that functional orthopedic therapy actively alters the natural condylar growth direction and rotational orientation [41]. Three-dimensional spatial analyses are consistent with a possible redirection of condylar growth by functional appliances, promoting a true anterior and inferior displacement relative to the cranial base while simultaneously modifying natural axial rotation [36,40,41]. Furthermore, Cevidanes et al. demonstrated that the more anterior alignment of the mandibular rami, measured via Procrustes geometric transformations, exhibited highly significant differences (*p* < 0.001) compared to untreated controls [36]. Across the eight included studies, seven evaluated condylar position as a geometric outcome. Specifically, Chintakanon et al., Chavan et al., Jiang et al., Elfeky et al., Arat et al., and Arici et al. [37,39,40,41,43] assessed condylar position primarily using a condyle–fossa reference (joint spaces, volumetric joint spaces, or concentricity-type indices), as previously described, whereas Cevidanes et al., Jiang et al., and Elfeky et al. [36,40,42] evaluated condylar positional changes as three-dimensional displacements relative to cranial reference structures, and Franco et al. [38] focused on articular disc position and morphology rather than condylar position. Quantifying anterior and inferior spatial displacement via CBCT, Elfeky et al. recorded a significant forward translation of both the right (Net Effect 1.50 mm, *p* < 0.001) and left (Net Effect 1.30 mm, *p* < 0.001) condyles relative to a stable vertical cranial plane [40]. Concurrently, the condyles exhibited a significant downward displacement relative to the Frankfort horizontal plane, measuring 0.53 mm (*p* = 0.046) on the right side and 0.59 mm (*p* = 0.039) on the left side. In conclusion, most studies consistently indicate a tendency for the condyle to assume a more anterior position within the glenoid fossa, or to translate anteriorly relative to the cranial base, following functional appliance therapy, suggesting a condylar advancement effect associated with joint remodelling phenomena. An exception is the study by Arici et al. [43], which reported posterior condylar positioning following treatment with the fixed functional Forsus appliance, based on CT-derived volumetric joint space changes. (Table 3) [38].

• Clinical Outcomes

In all studies reporting clinical data, the original authors stated that no patient developed signs or symptoms of TMD at the end of treatment. However, none of the included studies employed standardized diagnostic instruments to quantify clinical TMD outcomes, and these observations were reported in narrative form. In some cases, such as in Franco et al., an improvement in disc morphology was observed, with transitions from pathological to physiological positions. Overall, the findings suggest that functional orthodontic treatment induces significant TMJ changes, mainly affecting the position and morphology of the condyle, without clinically detectable adverse effects.

#### 3.2.2. Other Outcomes

• Glenoid Fossa Modification

The analysis of the available literature shows that changes in the glenoid fossa following functional appliance therapy are less consistent than those observed in the condyle. Only a limited number of studies directly evaluated the fossa, employing different methodologies. Arici et al., using CT, reported a difference in glenoid fossa volume increase of 83 ± 27 mm^3^ in the treated group compared with a difference of 75 ± 26 mm^3^ in controls; however, this change lacked statistical significance (*p* = 0.713), suggesting that physiological growth may outweigh appliance-specific effects [43]. Jiang et al., through CBCT and voxel-based superimposition, documented evidence of morphological remodelling, rather than sheer enlargement, predominantly in the superior regions of the fossa, although no direct volumetric measurements were provided, interpreting it as an active remodelling process adapting to condylar displacement and overgrowth [42]. Similarly, spatial repositioning was detected by Elfeky et al., who used CBCT landmarks to demonstrate mildly significant dimensional alterations in the relationship between the most superior aspect of the mandibular fossa and specific cranial reference planes (e.g., Frankfort Horizontal plane, *p* = 0.019) following Twin Block therapy [40]. Chintakanon et al., also using MRI, analyzed the articular eminence and reported no clear evidence of remodelling [41]. Other studies (Arat et al., Franco et al., Cevidanes et al., Chavan et al.) did not include direct assessments of the fossa [36,37,38,39]. In summary, current evidence suggests that the glenoid fossa is more likely to undergo localized remodelling than true volumetric enlargement, with findings varying according to the imaging modality employed (CT/CBCT vs. MRI) and the observation period. These changes should therefore be interpreted as adaptive phenomena secondary to condylar repositioning, rather than as a direct and consistent effect of functional therapy (Figure S1).

• Condyle

Most CBCT and CT studies reported a volumetric and dimensional increase in the condyle in response to functional treatment [40,42,43]. Specifically, Elfeky et al. found a significant bilateral increase in condylar length, width, and height [40]. Condylar length demonstrated a net increase of 1.28 ± 0.16 mm on the right (*p* < 0.001) and 1.60 ± 0.16 mm on the left (*p* < 0.001). Furthermore, condylar width exhibited a net increase of 0.88 ± 0.16 mm on the right (*p* < 0.001) and 0.53 ± 0.17 mm on the left (*p* < 0.01), whereas condylar height increased by a net value of 1.59 ± 0.23 mm on the right (*p* < 0.001) and 1.10 ± 0.26 mm on the left (*p* < 0.01) compared with untreated subjects. Jiang et al. documented a significant absolute increase in condylar volume from 1399.73 ± 47.71 mm^3^ to 1613.42 ± 64.12 mm^3^ in treated patients, compared with an increase from 1454.11 ± 107.38 mm^3^ to 1509.79 ± 109.51 mm^3^ in controls (F = 30.78, *p* = 0.000) [42]. This volumetric expansion was accompanied by a significantly greater absolute increase in condylar superficial area, which expanded from 786.44 ± 18.84 mm^2^ to 860.41 ± 24.14 mm^2^ in the treated group, compared to a physiological increase from 784.12 ± 37.37 mm^2^ to 805.96 ± 37.66 mm^2^ in the control group (F = 27.53, *p* = 0.000). Arici et al. also reported an increase in condylar osseous volume in patients treated with the Forsus fixed functional appliance, from 318 ± 57 mm^3^ at T0 to 357 ± 68 mm^3^ at T1 [43]. However, because the control group in Arici et al. showed a very similar physiological volumetric increase from 314 ± 57 mm^3^ to 342 ± 60 mm^3^, the difference between the two groups did not reach statistical significance (*p* = 0.301). Overall, CBCT and CT studies consistently demonstrate that the condyle exhibits pronounced dimensional changes, although the statistical significance of volumetric enlargement may vary depending on the appliance used [40,42,43].

• Mandibular Ramus

Studies evaluating the mandibular ramus consistently reported an increase in its dimensions and spatial anterior alignment following functional appliance therapy [36,40]. In a cone-beam computed tomography (CBCT) controlled study involving 40 subjects, Elfeky et al. observed that the ramus length (Co-Go) showed a statistically highly significant net increase of 3.47 ± 0.41 mm (*p* < 0.001) compared with untreated controls [40]. Magnetic resonance imaging (MRI) studies provided three-dimensional morphological and spatial data rather than linear measurements regarding the ramus [36]. Specifically, using 3D MRI and Procrustes superimpositions on a sample of 78 subjects, Cevidanes et al. reported that deformation grids in the treated group showed a significantly more advanced anterior alignment and an increased vertical dimension of the entire mandibular ramus compared with both untreated Class II controls (*p* < 0.001) and normal occlusion subjects (*p* < 0.001) [36]. Collectively, the mandibular ramus emerges as one of the main skeletal sites of response, presenting both measurable linear elongation and favourable spatial reorientation [36,40].

• Mandibular Body

Although more limited, available evidence suggests a significant increase in mandibular body length in response to functional therapy [40]. In their CBCT analysis, Elfeky et al. reported a highly significant net linear increase of 2.69 ± 0.38 mm in the mandibular corpus length (Go-Gn) in the treated group compared with untreated controls (*p* < 0.001) [40]. Through 3D relational analysis, Cevidanes et al. described a more favourable overall mandibular growth orientation in treated patients, reflecting a biologic adaptation of the mandible as a whole, although specific linear quantification of the corpus length was not provided [36].

### 3.3. Risk of Bias and Study Quality

**Figure 2:**
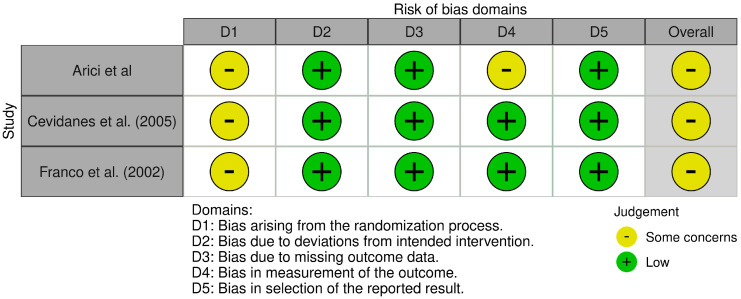
The risk of assessment bias for RCT studies [36,38,43].

**Figure 3:**
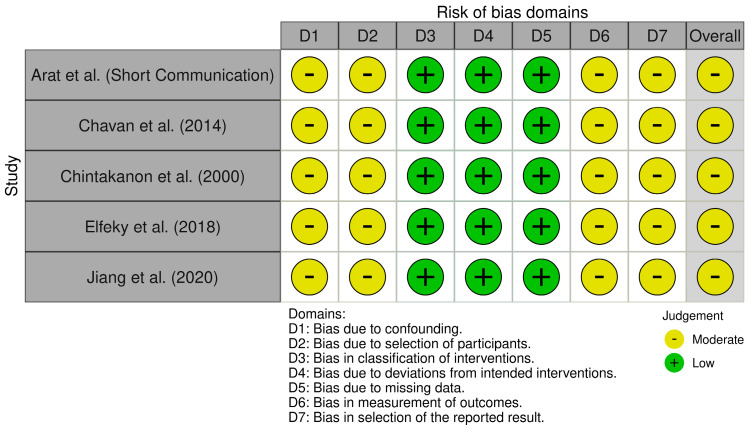
The risk of assessment bias for CCT studies [37,39,40,41,42].

The methodological quality of the included studies was assessed using tools appropriate for each study design. For RCTs, the Cochrane Risk of Bias 2 tool (RoB 2) was applied [33], whereas the ROBINS-I tool was used for non-randomized prospective studies [34]. The assessment focused on five domains: the randomization process, deviations from intended interventions, missing outcome data, measurement of the outcome, and selection of the reported result. Overall, the three RCTs included in this review (Arici et al., Cevidanes et al., Franco et al.) were judged as having ‘Some concerns’ [36,38,43]. While all three studies employed randomization, the specific methods of sequence generation and allocation concealment were often not detailed (Domain 1). Blinding of participants and personnel was not feasible due to the nature of the orthodontic interventions; however, this was not considered a critical source of bias for the objective imaging outcomes. As the primary outcomes are objective imaging-based measurements (linear, angular, volumetric), what is methodologically relevant is blinding of the outcome assessor rather than of participants; outcome assessors were blinded in most studies (Cevidanes et al., Franco et al.), thereby reducing detection bias (Domain 4) [36,38]. Attrition bias (Domain 3) and reporting bias (Domain 5) were generally low across the included trials. The risk of bias for the included non-randomized CCTs was assessed using the ROBINS-I tool (Risk of Bias In Non-randomized Studies-of Interventions) [34]. Overall, the studies (Arat et al., Chavan et al., Chintakanon et al., Elfeky et al., Jiang et al.) were judged to have a ‘Moderate’ risk of bias [39,40,41,42]. Due to the non-randomized nature of these investigations, confounding (Domain 1) was the primary concern, as baseline differences between treatment and control groups could not be entirely ruled out, although most studies attempted to match subjects by age and skeletal maturity. The selection of participants (Domain 2) was also rated as ‘Moderate’ risk, given the retrospective or prospective selection based on specific malocclusion criteria rather than random allocation. Risks associated with the classification of interventions (Domain 3) and deviations from intended interventions (Domain 4) were generally ‘Low’, as the treatment protocols were clearly defined. Missing data (Domain 5) was minimal across the studies, resulting in a ‘Low’ risk judgement. However, for the measurement of outcomes (Domain 6), a ‘Moderate’ risk was assigned where blinding of the outcome assessors was not explicitly confirmed, introducing potential detection bias in the analysis of imaging data. Finally, regarding the selection of the reported result (Domain 7), the risk was considered ‘Moderate’ due to the absence of pre-registered protocols, although the reported outcomes were consistent with the study objectives (Figure 2 and Figure 3).

### 3.4. Evidence Quality

The overall quality of the evidence was assessed using the GRADE (Grading of Recommendations Assessment, Development and Evaluation) approach [35]. The overall certainty of the evidence was assessed using the GRADE approach (Grading of Recommendations Assessment, Development and Evaluation). Despite the inclusion of three RCTs, the quality of evidence for the main outcomes (condylar position and morphology changes) was graded as “Low” to “Very Low” (Table S5). This assessment is based on the following factors:

- Risk of Bias: The included RCTs presented “some concerns” regarding randomization details and allocation concealment (Arici et al.; Cevidanes et al.; Franco et al.) [36,37,38]. The non-randomized studies presented a “moderate” risk of bias due to confounding and selection factors.
- Inconsistency: There was heterogeneity in the reported outcomes, particularly regarding condylar positional changes. While most studies reported an anterior or superior condylar movement, Arici et al. reported a posterior movement, likely due to differences in appliance mechanics (fixed vs. removable).
- Imprecision: The sample sizes in the included studies were relatively small (ranging from 18 to 78 participants), which limits the precision of the estimated effects and the power to detect significant differences.

Consequently, while the evidence suggests that functional appliances induce adaptive changes in the TMJ, the specific nature and magnitude of these changes should be interpreted with caution due to the limitations in the available evidence (Table S6).

## 4. Discussion

This systematic review was conducted to evaluate, using three-dimensional imaging techniques (CBCT or MRI), the morphological and positional changes of the articular disc and the temporomandibular joint (TMJ) in growing patients with Class II malocclusion treated with functional appliances. Class II malocclusion is one of the most common orthodontic alterations, whose genesis is frequently attributable to a skeletal retrusion of the mandible [4]. A thorough understanding of the anatomy and morphology of the temporomandibular joint (TMJ) in these patients, when compared to the normative parameters of Class I subjects, is crucial for formulating a treatment plan and assessing the susceptibility to temporomandibular disorders (TMDs) [44]. The mandibular condyle in normo-occlusive Class I patients is typically positioned centrally and concentrically within the glenoid fossa [45]. In untreated patients with Class II malocclusion, it has historically been thought that the condyle and mandible were retruded following an anteriorized head posture [46]. Recent studies have instead confirmed that the position of the condyle within the glenoid fossa in Class II division 1 is neither concentric nor posteriorized, but is generally translated in an anterior and medial direction [25,47], with three-dimensional hypoplasia of the condyle in all surface and volume measurements compared to Class I [4,44]. Our systematic review partially confirms this pattern, but above all, it confirms the individual variability in condylar positioning. In Chintakanon et al., within the entire sample of 40 children, 50% of the condyles were in an anterior position, 22.5% in a concentric position, and 27.5% in a posterior position. Chavan et al. reported that the sample analyzed in their study presented 60% of the condyles in a more anterior position within the fossa, although the values fell within normal physiological ranges. Jiang et al. analyzed 26 patients divided into two groups: 9 in the control group and 17 in the treatment group, both with Class II division 1. In the control group, the mean Joint Space Index (JSI) was 18.55 (indicating an anterior tendency), whereas in the group allocated to the Twin-block, the mean JSI was −0.55, denoting a concentric or anterior, but not posteriorized, pre-treatment position. The data from Elfeky et al. confirm a substantially balanced (concentric) position, with the anterior joint space (AJS) at approximately 2.39–2.45 mm and the posterior joint space (PJS) at 2.58–2.63 mm, demonstrating that the condyle is not retruded [40]. Interindividual variability is highlighted by the study of Arat et al., where the group allocated to treatment coincidentally had a larger anterior space than the posterior one (AJS 2.59 mm versus PJS 1.99 mm), while the control group, also in Class II division 1, presented the opposite situation (AJS 1.66 mm versus PJS 2.92 mm) [37]. The only exception is represented by the study of Arici et al. Through volumetric CT measurements, they showed that the anterior space (AJS) was physically larger than the posterior space (PJS) before treatment [43]. In pre-treatment patients allocated to the Forsus appliance, the initial AJS was 177 ± 43 mm^3^, compared to a PJS of 150 ± 42 mm^3^. In patients belonging to the pre-treatment control group, the AJS was 179 ± 45 mm^3^ and the PJS was 153 ± 43 mm^3^, indicating that the condyle starts from a more retruded position. The authors assume that the differences compared to other studies stem from methodological discrepancies: Arici et al. emphasize that in the traditional literature, the condyle–fossa relationship has always been evaluated by measuring the linear distance (in millimetres) or angles between two reference points on single sagittal scans (as Arat, Chavan, and Chintakanon did), whereas the latter utilized Computed Tomography (CT), extrapolating three transversal slices to calculate the true three-dimensional volume (in mm^3^) of the joint spaces using Cavalieri’s mathematical principle [37,39,41,43]. Therefore, it is not the condyle within the fossa that is retruded; rather, recent studies have highlighted that the entire glenoid fossa is positioned significantly more distally in Class II patients [25,48]. This difference is more clearly visible in Class II subdivision patients, where the glenoid fossa on the Class II side is positioned more distally and laterally compared to the Class I side and the anterior cranial base [49]. Regarding joint volumes, some studies highlight that patients with a Class I skeletal relationship present a greater total joint space compared to those with Class II and a clear tendency towards morphological contraction of the cavity in patients with mandibular retrusion, whereas others find this volumetric difference only when compared to Class II division 2 patients [25,50,51]. These discrepancies severely affect the biomechanical environment, configuring in Class II subjects glenoid fossae that are on average more distal, volumetrically reduced, and structurally altered in width and depth ratios compared to normo-occlusive patients [52]. These morphological characteristics have led to Class II malocclusion and mandibular retrognathia being associated with TMJ pathologies, particularly anterior disc displacement [11,43]. Studies have shown a significantly higher association with disc displacement, demonstrating an incidence of disc displacement without reduction (DDwoR) compared to Class I patients, and an incidence of displacement that in some studies reaches up to 53% of Class II cases [11,19,21,47,53]. The Class II skeletal pattern and the peculiar stress distribution on the condyle, which is vectorially positioned more anteriorly compared to Class I subjects, is therefore a factor that exposes patients to a significantly higher risk of developing anterior disc displacement compared to other craniofacial conformations [11,15,54]. Because of these characteristics, functional mandibular advancement appliances have been viewed with concern, under the assumption that by advancing the condyle within the fossa in structurally predisposed patients, they could increase the risk of articular disc displacement. Zhou et al. explain how, in cases of DDwR, there is a statistically significant increase in the anterior joint space and a drastic reduction in the posterior joint space compared to joints with a normally positioned disc [54]. The findings of the present review do not support this concern. The analyzed data provide a preliminary indication relevant to the initial clinical question: functional therapy was associated with imaging-documented remodelling of the articular structures, predominantly involving the condyle and the glenoid fossa. In the four studies that directly assessed the articular disc, no treatment-induced disc displacement was reported within the observation period; conclusions regarding the absence of TMD onset are limited by the lack of standardized clinical assessment in the included studies and by the short follow-up durations. The analysis of five studies agrees that propulsion appliances, including the Twin Block, Bionator, and Activator, reposition the condyle to a significantly more anterior location within the glenoid fossa. Arat et al. concluded that the activator allows for safe functional adaptation without any risk or symptoms of joint disorders [37]; Chavan’s study reached similar conclusions for the Bionator [39]. Regarding the Twin Block, sagittal concentricity measurements confirmed a highly significant anterior movement (*p* < 0.01) compared to pre-treatment data and the control group; this is supported by the measurements of Chintakanon et al., who concluded that in 75% of cases successfully treated with the Twin Block at 6 months, the condyle occupies a stably more anterior position compared to baseline observation [41]. Jing et al. show a clear advancement of the mandible [42]. Morphometric evaluations using magnetic resonance imaging indicate that this translation derives from a significantly more advanced anterior alignment (*p* < 0.001) of the entire mandibular ramus relative to the cranial base, in stark contrast to the mere positional maintenance observed in the untreated group [36]. Conversely, only one study concludes that the continuous and elastic force of the fixed Forsus appliance alters the proportions by pushing the condyle toward a posterior position. Functional orthopedic treatment may not be limited to inducing a temporary forward posture of the mandible, as the included studies suggest a possible stimulation of osteogenic activity compared to the physiological growth recorded in control groups, although this interpretation should be confirmed by further research. As a single divergence within the analyzed literature, the use of the fixed and inherently flexible Forsus appliance does record an increase in condylar bone volume from an initial 318 ± 57 mm^3^ to a final 357 ± 68 mm^3^, but this jump does not achieve true statistical significance (*p* = 0.301) when compared to the physiological increase of 28 ± 27 mm^3^ measured over the same 7 months in the untreated Class II group [43]. The anatomical changes of the glenoid fossa following the use of functional appliances are less constant than the condylar ones, primarily configuring as localized phenomena of spatial adaptation rather than a true expansion. The available data suggest that the glenoid fossa does not massively widen or hyperexpand as a direct and constant reaction to functional therapy but adapts locally through selective resorption (frequently superior) to tolerate the anterior displacement and the new joint dynamics [42]. In the four studies that directly assessed the articular disc with MRI, no new anterior disc displacements (ADD) were reported following treatment within the assessed follow-up; in selected cases, and an improvement of the pre-existing condyle–disc morphology was observed [38]. Within the limits of the available evidence, which was rated as Low to Very Low certainty according to GRADE, functional appliance therapy in growing skeletal Class II patients appears to be associated with adaptive condylar and joint remodelling, with no signal of increased risk of joint pathology emerging from the included controlled studies. These observations should be considered preliminary and require confirmation by adequately powered, long-term controlled trials before being translated into firm clinical recommendations.

In the limited subset of patients with anterior disc displacement with reduction (ADDwR) included in the available studies, functional treatment was not associated with worsening of the condition; however, the available evidence is too limited to support firm conclusions on a possible favourable effect on regenerative [38]. However, the stability of the disc position suggests that the primary objective should be skeletal and occlusal correction, rather than the “recapture” of the disc, which occurs unpredictably. Furthermore, the increase in the vertical dimension of the mandibular ramus and the overall length of the mandible is consistent with a possible effect of these devices in improving profile esthetics in growing patients [40].

### 4.1. Comparison with Existing Literature

The findings emerging from this review are positioned within a broad scientific debate, providing robust confirmations as well as important methodological distinctions compared with previous investigations. In strong agreement with the most recent systematic reviews, our data confirm that functional mandibular advancement does not cause iatrogenic damage to the TMJ and does not predispose growing patients to the development of TMDs [27]. The overall clinical conclusions of the present review are broadly consistent with those of other contemporary systematic reviews. The present work differs from these previous syntheses in several methodological aspects. Specifically, the present review included only primary controlled clinical studies, excluding previous reviews as sources of evidence, in order to avoid the overlap of samples and diagnostic heterogeneity that may arise when reviews are nested within reviews, as discussed by Ding et al. (2022) [27]. The inclusion criteria were restricted to studies employing three-dimensional imaging modalities (CBCT, CT, or MRI) in order to limit the well-known constraints of superimposition and anatomical distortion intrinsic to two-dimensional radiographic techniques, as previously highlighted in the literature [29]. No restrictions on language or year of publication were applied, and all functional appliances were considered eligible [55]. The inclusion of an untreated control group was required as a methodological criterion, allowing the observed changes to be interpreted relative to physiological growth [19]. Finally, a defined age range (8–16 years) was adopted to reduce demographic variability across the included samples [56]. A direct study-by-study comparison with the corpus of previous systematic reviews was not undertaken, as it falls outside the scope of the present synthesis.

### 4.2. Limitations of Review and Included Studies

This review presents intrinsic limitations related to the nature of the included primary studies. Many of the analyzed studies feature small sample sizes, often fewer than 30 subjects per group, which reduces the statistical power of the analyses. Moreover, most studies have a follow-up limited to the end of the active treatment phase or a few months thereafter, preventing the evaluation of the long-term stability of the observed remodelling and the assessment of TMD onset in adulthood. Methodologically, although controlled studies were included, some present risks of bias linked to the lack of randomization or unclearly defined blinding protocols.

### 4.3. Rationale for Narrative Synthesis

A quantitative meta-analysis was not undertaken in the present review. This methodological choice deserves explicit discussion, as it directly affects the interpretation of the findings. Following data extraction, substantial clinical and methodological heterogeneity emerged across the included studies. Five different functional appliances were employed (Andresen Activator, Twin Block, Bionator, Fränkel Regulator II, Forsus), each characterized by distinct biomechanical principles. Imaging modalities included MRI, CBCT, and conventional CT, with non-comparable spatial resolution and tissue specificity, and with MRI being the only modality capable of directly visualizing the articular disc. Treatment durations and follow-up intervals also varied, from 6 to 18 months. The narrative synthesis approach, conducted in accordance with the SWiM reporting guideline, was therefore preferred.

### 4.4. Recommendations for Future Research

In light of the highlighted gaps, future research should be directed towards the following:

- Multicenter RCTs with larger sample sizes to increase statistical power and reduce selection bias;
- Long-term follow-up: It is essential to monitor patients well beyond the end of functional therapy (at least 2–5 years post treatment, and ideally into adulthood) to confirm whether condylar and positional changes are permanent or subject to relapse, and to assess the potential onset of TMD signs and symptoms over time;
- Standardization of imaging protocols;
- Stratified subgroup analysis: Further studies should specifically investigate the treatment response in patients with different degrees of pre-existing disc displacement (ADDwR vs. ADDwoR), as suggested by recent works by Wang et al. (2025), to define personalized therapeutic protocols [57].

## 5. Conclusions

Based on the currently available studies, low-to-moderate evidence suggests that, in growing patients with skeletal Class II malocclusion, functional appliance therapy is associated with TMJ changes largely consistent with adaptive remodelling, with many reports indicating a tendency toward a more anterior condylar position and signs of condylar and/or glenoid fossa remodelling. Available data on soft tissue components generally indicate the stability of articular disc position, with no consistent evidence of treatment-induced disc displacement, and no clear increase in TMD signs/symptoms within the assessed follow-up. However, these conclusions should be interpreted with caution due to heterogeneity in imaging protocols and outcome measures, frequently small sample sizes, and limited follow-up durations. Further well-designed prospective controlled studies (ideally randomized when feasible) using standardized 3D outcomes and longer follow-up are warranted.

## Figures and Tables

**Table 1:** This table shows the inclusion and the exclusion criteria applied.

Inclusion Criteria	Exclusion Criteria
Human studies involving skeletal Class II patients with mandibular deficiency	Studies on patients with temporomandibular disorders (TMD) or syndromes
Treatment with functional appliances	Animal studies and in vitro studies data
Imaging with CBCT or MRI pre- and post-treatment	Case reports, reviews, editorials, case series, or opinion pieces
Studies focusing on TMJ changes	Cross-sectional studies, case–control studies, and studies without longitudinal pre- and post-treatment design
Prospective controlled clinical studies with a longitudinal pre–post design (RCTs and CCTs)	
All languages	
Age: 8–16 years	

**Table 2:** The main characteristics of the included studies (TB = Twin Block; FR-II = Fränkel Regulator II; FT/NT = functional-treatment vs. no-treatment groups as defined in the original study; CCT = controlled clinical trial).

Author and Year	Study Design	Appliance	Imaging	Treated Group	Control Group	Dx Criteria	Treatment Duration	Follow-Up
Arat et al. 2001 [37]	CCT	Andresen Activator	MRI	9 Class II	9 untreated Class II	ANB > 4°, GoGn–SN 25–32°, increased overjet/overbite, mandibular retrusion	16 months (mean)	6 months
Franco et al. 2002 [38]	RCT	Fränkel Regulator II (FR-II)	MRI	28 Class II	28 untreated Class II	ANB ≥ 4°, retrognathic mandible, normal transverse relations	18 months	18 ± 1 months
Chavan et al. 2014 [39]	CCT	Twin Block (n = 10), Bionator (n = 10)	MRI	20 Class II 10 TB 10 Bionator	10 untreated Class II	ANB > 5°, overjet ≥ 6 mm, retrognathic mandible	6 months	6 months
Elfeky et al. 2018 [40]	CCT	Twin Block	CBCT	22 Class II	18 untreated Class II	ANB > 5°, overjet ≥ 6 mm	9.4 ± 1.3 months	8 months
Chintakanon et al. 2000 [41]	CCT	Twin Block	MRI	19 Class II	21 untreated Class II	Retrognathic mandible, short lower facial height, overjet > 5 mm	6 months	6 months
Cevidanes et al. 2005 [36]	RCT	Fränkel Regulator II (FR-II)	MRI	28 Class II	25 untreated Class II + 25 normal Class I	ANB 4.5–10 mm, ≥¾ cusp Class II molars, overjet 4.5–10 mm	18 months	18 ± 1 months
Jiang et al. 2020 [42]	CCT	Twin block	CT	17 Class II	9 untreated Class II	ANB > 5°, overjet > 5 mm, CVMI stage 3–4	8 months	8 months
Arici et al. 2008 [43]	RCT	Forsus (fixed functional)	CBCT	30 Class II	30 untreated Class II	Skeletal Class II Division 1, retrognathic mandible, overjet > 5 mm	7 months	7 months

**Table 3:** The imaging modality and reference system used to assess condylar position.

	Imaging	Reference
Chintakanon et al. [41]	MRI	Glenoid Fossa *Pullinger method*
Elfeky et al. [40]	CBCT	Glenoid Fossa + Cranial basis *Linear evaluation*
Arici et al. [43]	CT	Glenoid Fossa *Linear evaluation*
Jiang et al. [42]	CBCT	Glenoid Fossa *Joint Space Index*
Cevidanes et al. [36]	MRI	Cranial Basis *Linear evaluation*
Chavan et al. [39]	MRI	Glenoid Fossa *Pullinger method*
Franco et al. [38]	MRI	Not evaluated
Arat et al. [37]	MRI	Glenoid Fossa *Articular space analysis*

## Data Availability

The data presented in this study are available in the article.

## Supplementary Materials

The following supporting information can be downloaded at: https://www.mdpi.com/article/10.3390/children13050674/s1, Figure S1: showing which study analyzes the skeletal outcomes on condyle, mandibular ramus, body and glenoid fossa; Table S1: Search Strategy 1 for each database; Table S2: showing which study analyzes the main outcomes on disc and articular spaces; Table S3: Summary of treatment articular disc position and morphology measurements following functional orthopedic therapy; Table S4: Summary of temporomandibular joint (TMJ) spaces and condylar position changes following functional orthopedic treatment. Table S5: showing the grade of evidence. Table S6: summarizes the quality of evidence for each outcome, highlighting the main strengths and limitations that influenced the certainty grading.
