# Supplementary material for: Temporomandibular Joint Changes Assessed by CBCT or MRI Following Functional Appliance Therapy in Skeletal Class II Patients: A Systematic Review
Source: Children (Basel). 2026 May 13;13(5):674. doi: 10.3390/children13050674 (PMC13204457; doi:10.3390/children13050674)
Supplement: Supplementary file 1 [file children-13-00674-s001.zip › children-4261697-supplementary.pdf]

Database	Search Strategy
PubMed	(("magnetic resonance imaging"[MeSH Terms] OR "MRI" OR "CBCT") AND ("temporomandibular joint"[MeSH Terms] OR "TMJ") AND ("functional appliance" OR "orthodontic" OR "activator" OR "Herbst" OR "Frankel" OR "twin block" OR "Forsus")
Scopus	(TITLE-ABS-KEY ("magnetic resonance imaging" OR mri OR cbct) AND TITLE-ABS-KEY ("temporomandibular joint" OR tmj) AND TITLE-ABS-KEY ("functional appliance" OR orthodontic OR activator OR herbst OR frankel OR "twin block" OR forsus))
Cochrane Library	("magnetic resonance imaging" OR MRI OR CBCT) AND ("temporomandibular joint" OR TMJ) AND ("functional appliance" OR "orthodontic" OR Herbst OR "twin block" OR Forsus OR Frankel OR "mandibular advancement")
Web of Science	TS=("magnetic resonance imaging" OR MRI OR CBCT) AND TS=("temporomandibular joint" OR TMJ) AND TS=("functional appliance" OR orthodontic OR activator OR Herbst OR Frankel OR "twin block" OR Forsus))
Embase	('nuclear magnetic resonance imaging'/exp OR 'nuclear magnetic resonance imaging' OR 'cone beam computed tomography'/exp OR 'cone beam computed tomography') AND ('temporomandibular joint'/exp OR 'temporomandibular joint' OR TMJ) AND ('functional appliance'/exp OR 'functional appliance' OR 'orthodontic' OR 'herbst appliance'/exp OR 'herbst appliance' OR frankel OR forsus OR 'twin-block appliance'/exp OR 'twin-block appliance')

Table S1: *Search Strategy 1 for each database*

	Disc Morphology	Disc Position	Articular Spaces
Franco et al.(2002)	Improvement	Stable	Not analized
Arat et al. (2001)	Not statistically significant	Stable	yes
Chavan et al. (2014)	Not analized	Improvement	yes
Chintakanon et al. (2000)	Not analized	Stable	yes
Elfeky et al. (2018)	Not analized	Not analized	yes
Cevidanes et al. (2005)	Not analized	Not analized	Not analized
Jiang et al. (2020)	Not analized	Not analized	yes
Arici et al. (2008)	Not analized	Not analized	yes ≠

Table S2: *showing which study analyzes the main outcomes on disc and articular spaces.*

Study	Sample (N)	Appliance	Imaging	Measurement Method	Pre/Post-Treatment Disc Measurement and Difference	Pre/Post Control Patients Disc Measurement and Difference	Statistical Significance	Critical Explanation
Chintakanon et al, 2000	40 children (19 treated, 21 controls); 40 TMJs (Right only)	Clark Twin-block	MRI (1.0-T)	Sagittal position (angles relative to 12 o'clock using FH and PC lines); Closed mouth.	relative to PC-line:<br>- Anterior margin post: 114.4 ± 7.3; D -3.2 ± 7.3<br>- Posterior margin: 19.5 ±11.3; **D -7.6 ± 13.4**<br>Relative to FH-line:<br>- Anterior Margin: 94.5±7.8; D: -3.5±9.0<br>- Posterior Margin: -0.2±13.8; D: -3.3±8.7	Relative to PC-line:<br>- Anterior margin post: 114.0 ± 10.3°; D: -1.7 ±6.4<br>- Posterior margin post: 19.2 ± 10.4; D: -3.6 ±12.6<br>Relative to FH-line:<br>- Anterior Margin: 93.3±9.1; D -7.1±8.5<br>- Posterior margin: -1.7 ±10.7; D: -6.5±9.3	Disc appeared to move more posteriorly in both groups over 6 months; no evidence of disc recapture.<br><br>P results:<br>- Treated Posterior margin (PC-line) Pre/Post: *P*<0.05<br>- Control (FH-line) Pre/Post: *P*< 0.01<br><br>P<0.05 SS (statistically Significant)	The authors noted that this depended on the reference line used (Frankfort plane vs posterior condylar line). The apparent shift was likely a growth-related reference-orientation artifact rather than a true anatomical disc displacement.
Chavan et al, 2014	30 subjects (10 Twin Block, 10 Bionator, 10 control); 30 TMJs (Right only)	Twin Block + Bionator	MRI (0.2-T)	Sagittal disc position measured as an angle relative to the 12 o'clock position (intersecting line through condylar center); Closed mouth	- Twin Block Post: 1.8±0.2; D: -19.4±9.1.<br>- Bionator Post: −0.9±0.5; D: -16.4±11	- 8.6±10.9; D: -2.6±0.9	Disc moved more posteriorly in relation to the condyle in both treatment groups compared to pre-treatment.<br><br>P results:<br>- Control vs TB: P<0.01<br>- Control vs B: P<0.01<br>- TB vs B: P>0.05<br><br>P>0.05 NS<br>P<0.01 HS	
Arat et al, 2001	18 subjects (9 treated, 9 controls); 18 TMJs (Left only)	Andresen activator	MRI (1.5-T)	Angular measurements (Por-Dios program). Angles between condylar line and disc bands ;Closed mouth.	- Medial angle Post: 61.57±4.47; D: 5.27±4.33<br>- Posterior angle Post: −1.95±1.96; D: -0.26±3.70<br>- Anterior angle Post: 101.9±5.74; D: 3.28±5.51	- Medial angle Post: 53.01±3.22; D: 4.02±4.56<br>- Posterior angle Post: 0.57±1.87; D: 1.95 ±2.50<br>- Anterior angle Post: 94.4±3.63; D: 0.20 ± 5.57	No changes NS for all disc angle changes<br><br>*P result* >0.05<br><br>P>0.05 NS	The changes in posterior and anterior angles reflect a non-significant, physiological adaptation of the disc–condyle complex to the condyle's more

								anterior position in the fossa
Franco et al, 2002	56 children (28 treated, 28 controls); 112 TMJs	Fränkel Functional Regulator-II (FR-II)	MRI (0.5-T)	Qualitative classification of disc position (9 categories) in closed and open mouth; disc shape (biconcave vs nonbiconcave).	Closed mouth:<br>- 100% superior position; D: =<br>Open mouth:<br>- 100% interposed; D: =<br>Shape:<br>- 100% biconcave shape D: pre-treat. 10.7% non-biconcave	Closed mouth:<br>- 92.9% Superior; 7.1% Partial anterior medial displacement<br>Open mouth:<br>- 92.9% interposed;<br>- 1.8% displacement with reduction<br>- 5.3% displacement without reduction<br>Shape<br>- 82.1% biconcave<br>- 17.9%non-biconcave	No positional changes occurred. Disc morphology in the treated group significantly improved to 100% biconcave.<br>P results:<br>Shape P<0.05 (P =0.016)<br><br>P<0.05 SS	

Table S3: Summary of treatment articular disc position and morphology measurements following functional orthopedic therapy

B: Bionator appliance; **CTB:** Clark Twin-block appliance; **D:** Mean Difference (Post-treatment minus Pre-treatment / R2 - R1); **FH:** Frankfort Horizontal plane; **FR-II:** Fränkel Functional Regulator-II; **MRI:** Magnetic Resonance Imaging; **N:** Number of subjects/samples; **NS:** Not Significant; **PC:** Posterior Condylar line; **SD:** Standard Deviation; **SS:** Statistically Significant; **T:** Tesla (magnetic field strength); **TB:** Twin Block appliance; **TMJ:** Temporomandibular Join

Data are presented as Mean ± Standard Deviation (SD) unless otherwise specified

Study	Condylar volume/dimensions	Mandibular ramus	Mandibular body	Glenoid fossa
Elfeky 2018	✓↑	✓↑	✓↑	✗
Jiang 2020	✓↑	✗	✗	✓ (remod)
Arici 2008	✓↑ (NS)	✗	✗	✓↑ (NS)
Cevidanes 2005	✗	✓↑	✗	✗
Chintakanon 2000	✗	✗	✗	✓ (no changes)

- ✓↑ = aumento significativo
- ✓↑ **(NS)** = aumento non significativo
- ✓ **(remod)** = rimodellamento osservato
- ✓ **(no changes)** = analizzata ma senza modifiche
- ✗ = non analizzata

Figure S1 *: showing which study analyzes the skeletal outcomes on condyle, mandibular ramus, body and glenoid fossa*

Study	Method of Analysis	Treated Group (Post-treatment)	Control Group (Post-treatment)	Statistical significance	Clinical Meaning
Arat et al., 2001	Magnetic resonance imaging (MRI) with Por-Dios software program. Measurements of anterior, superior, and posterior joint spaces (mm)	- AJS (Ca–Ca'): 1.98 ± 0.23 mm; D = -0.59 ± 0.19 mm<br>- SJS (Cs–Cs'): 3.49 ± 0.20 mm D = 0.29 ± 0.31 mm<br>- - PJS (Cp–Cp'): 2.72 ± 0.23 mm D = 0.73 ± 0.22 mm	- AJS (Ca–Ca'): 1.72 ± 0.13 mm; D = 0.06 ± 0.27 mm<br>- SJS (Cs–Cs'): 3.29 ± 0.19 mm; D = -0.35 ± 0.31 mm<br>- - PJS (Cp–Cp'): 2.65 ± 0.16 mm; D = –0.28 ± 0.16 mm	- AJS: P<0.05<br>- PJS: P<0.01	Activator repositioned the condyle anteriorly within the fossa without significant disc displacement.
Arici et al., 2008	Transverse Computed Tomography (CT) images	- AJS volume: 244 ± 59 mm3; D = +67 ± 40 mm3<br>- PJS volume: 137 ± 41 mm3; D = -13 ± 31 mm3	- AJS volume: 216 ± 48 mm3; D = +37 ± 31 mm3<br>- - PJS volume: 156 ± 45 mm3; D = +3 ± 7 mm3	Forsus-control<br>- AJS volume P=0.021<br>- PJS volume P=0.013	The condyle is repositioned posteriorly in the glenoid fossa due to an increase in anterior joint space and a decrease in posterior joint space.
Cevidanes et al., 2005	3D Magnetic Resonance Imaging (MRI) with Procrustes geometric transformation and 3D skeletal landmarks.	- Condylion (rCo/lCo): **relative posterior and superior bending**<br>- Gonion (rGo/lGo): more anteriore and inferior<br>- Increased mandibular rami vertical dimensions<br>- More forward rami relative to middle cranial fossae	- Maintenance of landmark configuration with growth (little deformation	Treated Group<br>Highly significant skeletal alterations vs controls P <0.01<br>More forward rami relative to middle cranial fossae and nasomaxilla.<br><br>Control Group<br>NS maintanance of landmark	FR-II significantly redirects mandibular growth: the mandibular ramus as a whole shifts anteriorly relative to the cranial base while the condylar region remodels posteriorly and superiorly to maintain a stable anatomical relationship within the glenoid fossa.
Chavan et al., 2014	Magnetic Resonance Imaging (MRI). Sagittal concentricity formula: [(P-A)/(P+A)] x 100.	Twin-Block<br>18.7 ± 10.3%; D = +15.01 ± 7.9%<br><br>Bionator<br>19.1 ± 11.5%; D = +11.38 ± 7.1%	6.7 ± 4.9%; D = -0.17 ± 0.2%	Twin-Block<br>P<0.01 HS<br><br>Bionator<br>P<0.01 hs<br><br>Control<br>P>0.05 (P=-0.15)	The condyles occupy a more anterior position in the fossa after 6 months of functional therapy compared with the pre-treatment stage. Although not

					statistically significant (NS), patients treated with the Twin-block show a more anterior condylar position.
Elfeky et al., 2018	Cone Beam Computed Tomography (CBCT) with Anatomage software. 3D osseous evaluation of TMJ and joint spaces (mm).	Right<br>- AJS : 1.56 ± 0.72; D = -0.88±0.69<br>- SJS: 3.61 ± 0.92; D = 0.71±0.65<br>- RJS: 3.60 ± 0.66; D = 1.02±0.75<br>- MJS: 3.01±0.89; D = -0.63±0.18<br>- Cds-FHP: 0.68 ±1.4; D = -0.42±0.89<br>- Cda-VP: -1.11 ±3.46; D = 1.49±0.65<br>- Cdl-MSP: 47.18 ±2.47; D = 0.16±0.86<br>Left<br>- AJS: 1.44 ± 0.58; D = -0.94±0.58<br>- SJS: 3.78 ± 0.89; D = 0.84±0.84<br>- PJS: 3.78 ± 0.57; D = 1.15±0.81<br>- MJS: 3.1 ± 1.03; D = -0.75±0.32<br>- Cds-FHP: 0.36 ±1.45; D = -0.42±0.94<br>- Cda-VP: -4.67 ±2.80; D = 1.45±0.69<br>- Cdl-MSP: 45.60 ±2.68; D= -0.24±0.81	Right<br>- AJS:1.85 ± 0.49; D = -0.11±0.33<br>- SJS: 2.85 ± 0.65; D = -0.07±0.61<br>- PJS: 3.28 ± 0.77; D = -0.22±0.35<br>- MJS: 3.70±0.91; D = 0.02±0.34<br>- Cds-FHP: -2.13 ±0.86; D = 0.11±0.44<br>- Cda-VP: -4.90 ±2.28; D = -0±0.38<br>- Cdl-MSP: 45.45 ±1.80; D = 0.64±1.62<br>Left<br>- AJS: 1.81 ± 0.6; D = -0.10±0.31<br>- SJS: 3.27 ± 0.83; D =-0.05±0.45<br>- PJS: 3.36 ± 0.79; D = 0.03±0.27<br>- MJS: -0.08±0.36; D = -0.08±0.36<br>- Cds-FHP: -1.82 ±0.75; D = 0.17±0.46<br>- Cda-VP: -4.56 ±2.52; D = 0.15±0.46<br>- Cdl-MSP:45.28 ±3.17; D = -0.17±0.65	Treated Group (Net Effect):<br><br>Right<br>- AJS:0.77 ± 0.19; P<0.01<br>- SJS: -0.79 ± 0.21; P<0.01<br>- PJS: -0.80±0.21; P<0.01<br>- MJS: 0.65±0.10; P<0.001<br>- Cds-FHP: 0.53±0.25; P=0.046<br>- Cda-VP: -1.50±0.18; P <0.001<br>- Cdl-MSP: -0.62 ±0.44; P = 0.170<br><br>Left<br>- AJS: 0.84±0.16; P<0.001<br>- SJS: -0.90±0.23; P<0.001<br>- PJS: -1.11 ±0.22; P<0.001<br>- MJS: 0.67±0.12; P<0.001<br>- Cds-FHP: 0.59±0.26; P = 0.039<br>- Cda-VP: -1.30 ±0.20; P<0.001<br>- Cdl-MSP: 0.60±0.25; P=0.792<br><br>P≤0.05 MS<br>P≤0.01S<br>P≤0.001HS	The Twin Block produces an anterior and inferior repositioning of the condyle, as shown by increased posterior and superior joint spaces and reduced anterior joint space..
Jiang et al., 2020	Cone Beam Computed Tomography (CBCT). Voxel-based 3D superimposition and joint space index (JSI).	- AJS: 2.61 ± 0.17 mm;<br>- PJS: 3.38 ± 0.24 mm<br>- JSI: 11.42 ± 3.54	- AJS: 1.88 ± 0.10 mm<br>- PJS: 2.47 ± 0.14 mm<br>- JSI: 13.22 ± 3.43	Treated Group<br>- AJS: +0.42 mm; *P*=0.005<br>- PJS: +1.19 mm; *P*<0.001 (P=0.000)<br>- JSI: da – 0.55 a	Twin-block treatment produced a reposition of the condyle more forward

				11.42; $P<0.05$ ($P=0.004$)<br>Control Group<br>- PJS: -0.44 mm; $P=0.021$	
Chintakanon et al., 2000	Magnetic Resonance Imaging (MRI). Sagittal concentricity formula.	- Sagittal concentricity: 75% anteriorly positioned condyles (in successful cases)<br>- Condylar axial angle: stable (no significant change from R1)	- Sagittal concentricity: mixed (62% anterior, 24% concentric, 14% posterior)<br>- Condylar axial angle: 54.4 ± 8.9 degrees (reduction compared to R1)	**Treated Group:** Condylar position: 53% of cases showed a shift toward more anterior categories<br><br>**Control Group:** Condylar axial angle: significantly reduced by 4.1 degrees (natural growth).<br><br>Condylar position P=0.01 (for successful cases)	Condyles positioned anteriorly by Twin-block reseated back into the fossa but remained more anterior relative to pretreatment position

Table S4: *Summary of temporomandibular joint (TMJ) spaces and condylar position changes following functional orthopedic treatment.*

3D: Three-Dimensional; **AJS:** Anterior Joint Space; **Ca/Ca', Cs/Cs', Cp/Cp':** Anterior, Superior, and Posterior condylar and fossa reference points (according to Arat et al.); **CBCT:** Cone Beam Computed Tomography; **Cda-VP:** Anteroposterior condylar position (perpendicular distance between the anterior condylar point and the Vertical Plane); **Cdl-MSP:** Mediolateral condylar position (perpendicular distance between the medial condylar point and the Midsagittal Plane); **Cds-FHP:** Vertical condylar position (perpendicular distance between the superior condylar point and the Frankfort Horizontal Plane); **CT:** Computed Tomography; **D:** Mean Difference (Post-treatment minus Pre-treatment); **FHP:** Frankfort Horizontal Plane; **FR-II:** Fränkel Functional Regulator-II; **JSI:** Joint Space Index; **lCo / rCo:** Left Condylion / Right Condylion; **lGo / rGo:** Left Gonion / Right Gonion; **MJS:** Medial Joint Space; **MRI:** Magnetic Resonance Imaging; **MSP:** Midsagittal Plane; **PJS:** Posterior Joint Space; **SJS:** Superior Joint Space; **TMJ:** Temporomandibular Joint; **VP:** Vertical Plane

Units of Measurement:

- Linear measurements indicating joint spaces or distances are expressed in millimeters (mm).
- Volumetric measurements are expressed in cubic millimeters (mm^3).
- Angular measurements determining condylar or skeletal inclinations are expressed in degrees (°).
- Proportional and relative positional calculations (e.g., sagittal concentricity) are expressed in percentages (%)

Statistical Notes:

- Data are presented as Mean ± Standard Deviation (SD) unless otherwise specified.
- **NS:** Not Significant ($P>0.05$).
- **MS:** Mildly Significant / Statistically Significant ($P\leq0.05$).
- **S:** Significant ($P\leq0.01$).
- **HS:** Highly Significant ($P\leq0.001$).

Outcome	Key Findings (Impact of Treatment)	No of Participants (Studies)	Certainty of the Evidence (GRADE)
Articular Disc Position (MRI)	No adverse effect. Most studies found that functional appliance therapy did not induce anterior disc displacement (ADD) or other pathological positional changes. The disc-condyle relationship was generally maintained or improved. *Specifics:* Franco et al. found 0% new displacement in treated group. Arat et al. found insignificant changes.	144 (4 studies: Franco, Chintakanon, Chavan, Arat)	⊕◯◯◯ VERY LOW a, b, c
Articular Joint Spaces (MRI, CT)	Significant changes observed. Treatment alters joint spaces, reflecting condylar repositioning. Results vary by appliance: Removable appliances typically increased posterior joint space (anterior condylar movement), while fixed functional appliances (Forsus) increased anterior joint space (posterior condylar movement).	148 (4 studies: Arici, Arat, Chintakanon, Chavan)	⊕◯◯◯ VERY LOW a, b, c
Condylar & Fossa Morphology (CBCT, MRI, CT)	Adaptive remodeling confirmed. Evidence of increased condylar volume, surface area, and potentially glenoid fossa adaptation compared to controls. 3D analysis (CBCT/MRI) showed significant localized growth/remodeling in the superior and posterior condylar regions.	163 (4 studies: Arici, Cevidanes, Elfeky, Jiang)	⊕⊕◯◯ LOW a, c
Condylar Position (CBCT, MRI, CT)	Anterior/Superior repositioning (predominant). The majority of studies using removable appliances (Twin Block, Fränkel, Bionator) reported a more forward (anterior) and often superior position of the condyle in the fossa. *Exception:* Fixed appliances (Forsus) resulted in a posterior condylar position.	292 (7 studies, all included studies except Franco et al., which evaluated only the articular disc)	⊕◯◯◯ VERY LOW a, b, c
Clinical Risk of TMD (Signs & Symptoms)	No new clinical TMD signs or symptoms were reported by the original authors during the observation period. None of the included studies applied standardized clinical assessment tools (e.g., DC/TMD or RDC/TMD); clinical outcomes were reported in narrative form, without structured quantification.	152 (3 studies reporting clinical data: Franco, Chintakanon, Arat)	⊕◯◯◯ VERY LOW a, c

Table S5 : showing the grade of evidence.

RCT: Randomized Controlled Trial; CCT: Controlled Clinical Trial; TMJ: Temporomandibular Joint; MRI: Magnetic Resonance Imaging; CBCT: Cone Beam Computed Tomography; TMD: Temporomandibular Disorders. Explanations for downgrading: a. Risk of Bias: Downgraded by one level because the included RCTs presented "some concerns" regarding randomization details (e.g., unclear allocation concealment mechanisms) and the non-randomized studies presented a "moderate" risk of bias due to potential confounding factors. b. Inconsistency: Downgraded by one level due to heterogeneity in the reported outcomes. Specifically, results regarding joint spaces and condylar position varied significantly depending on the appliance mechanics (e.g., contrasting findings between fixed Forsus appliances and removable Twin Block/Fränkel appliances). c. Imprecision: Downgraded by one level due to relatively small sample sizes in the included studies (ranging from 18 to 78 par-ticipants), which limits the precision of the effect estimates

Outcome	Strength of Evidence & Critical Appraisal
Articular Disc Position	VERY LOW. Evidence suggests that functional appliance therapy does not induce pathological disc displacements.<br>• Strengths: Data include 3 RCTs (e.g., Franco et al.) confirming the safety of the treatment regarding disc position.<br>• Limitations: The overall sample size is small, and imaging methodologies vary across studies.
Articular Joint Spaces	VERY LOW. Results show significant but inconsistent changes across studies.<br>• Strengths: Objective quantitative measurements on MRI/CT.<br>• Limitations: High heterogeneity due to appliance type: removable appliances tend to increase posterior joint space (anterior condylar movement), whereas fixed appliances (Forsus, Arici et al.) tend to increase anterior joint space (posterior condylar movement).
Condylar & Fossa Morphology	LOW. There is emerging consensus that therapy stimulates adaptive remodeling (increased condylar/glenoid volume).<br>• Strengths: The use of 3D technologies (CBCT, 3D MRI in Cevidanes et al.) provides precise volumetric data less prone to distortion compared to 2D imaging.<br>• Limitations: Selection bias in non-randomized studies (CCTs) reduces overall certainty.
Condylar Position	VERY LOW. Although this is the most studied outcome, results are highly dependent on the mechanics used.<br>• Strengths: Large number of patients analyzed across studies.<br>• Limitations: Conflicting results (clinical heterogeneity): most studies report an anterior positioning, except for fixed continuous-force appliances which show a posterior positioning.
Clinical Risk of TMD	VERY LOW. Available clinical data indicate no new signs or symptoms of temporomandibular dysfunction post-treatment.<br>• Strengths: Consistency among the few studies reporting this data.<br>• Limitations: None of the included studies applied standardized clinical assessment tools (e.g., DC/TMD or RDC/TMD); clinical TMD outcomes were not systematically quantified, and follow-up periods are limited.

Table S6: summarizes the quality of evidence for each outcome, highlighting the main strengths and limitations that influenced the certainty grading.
